# Protocol for the development of a Core Outcome Set (COS) for reporting outcomes of clinical trials of anti-tuberculosis treatment in adults with pulmonary tuberculosis

**DOI:** 10.1186/s13063-025-09224-8

**Published:** 2025-11-18

**Authors:** Rebecca Thomas, Nicola L. Harman, Paula R. Williamson, Laura Bonnett, Geraint R. Davies, TB-COS Steering Committee, Patrick Phillips, Hanif Esmail, Daniela Cirillo, Susan Dorman, Christian Leinhardt, Jonathan Stillo, Oxana Rucsineanu, Blessina Kumar, Carrie Tudor, Katie Rolfe, Jeremiah Chakaya

**Affiliations:** Department of Health Data Science, University of Liverpool, Liverpool, UK

**Keywords:** Core outcome set, Tuberculosis, Pulmonary tuberculosis, Consensus, Delphi study, Treatment outcome, Clinical trial

## Abstract

**Background:**

The global burden of tuberculosis (TB) remains high, and treatment of both drug-susceptible and drug-resistant diseases is complex and prolonged. International clinical trial networks and public-private partnerships are beginning to deliver advances in TB treatment, and collaborative data-sharing efforts are being promoted to improve the transparency and scrutiny of the evidence used in formulating treatment guidelines and public health policies. Empirical studies have identified variability in the selection and reporting of clinical trial outcomes in historical and recent phase III trial reports and protocols in TB. This variability could affect the synthesis of evidence and interpretation by practitioners, regulators, and policymakers.

**Methods and analysis:**

A comprehensive list of outcomes used in phase III treatment trials of pulmonary TB in adults, grouped according to key domains, will be identified from systematic reviews of published trials and trial protocols. A systematic review of qualitative evidence will inform outcomes important to people diagnosed with TB. Consensus will be sought from an international panel of participants representing key stakeholder groups using a multi-stage Delphi process. The final COS identified from the Delphi survey will be ratified at an online consensus meeting and approved by the SSC. The process will be conducted according to the Core Outcome Set-STAndards for Development (COS-STAD) guidance and with the support of the Core Outcome Measures in Effectiveness Trials (COMET) Initiative.

**Discussion:**

A COS implemented in the TB trials community that reflects the outcomes most important to stakeholders would help ensure that important evidence is translated rapidly, clearly, and effectively into practice to maximise patient benefit.

**Trial registration:**

The study has been registered with the COMET database of core outcome sets (study 2195, registered October 2022).

## Introduction

### Background

Tuberculosis (TB) remains among the most lethal of infectious diseases, causing 1.25 million deaths each year and accounting for a third of lives lost due to antimicrobial resistance [24, 27, 28]. TB is caused by *Mycobacterium tuberculosis*, an obligate human pathogen with which 20–25% of people are estimated to be infected globally [18, 27, 28]. While the infection is asymptomatic, approximately 5% of infected people will develop active disease in their lifetime, resulting in a huge burden of 10 million disease episodes each year. Active disease is most often pulmonary TB (PTB) but can be extrapulmonary TB (EPTB) or severe forms such as disseminated, meningeal, or pericardial TB. The burden of TB is disproportionately concentrated in 49 high-burden countries, which account for at least 86% of cases [27, 28]. Significant barriers to care remain in many high-burden countries [27, 28]. Around a third of people with drug-susceptible TB (DS-TB) and two-thirds of those with drug-resistant TB (DR-TB) are not diagnosed by the health system, while for those who are, treatment success rates are 88% and 68%, respectively [27, 28].

Among bacterial pathogens, treatment of TB is uniquely complex and prolonged, requiring regimens comprising up to 7 drugs for as long as 24 months, depending on patterns of resistance [29]. After a 30-year hiatus, renewed efforts in drug development and increasing clinical trial activity have recently led to incremental advances in the treatment of both DS-TB and DR-TB. Treatment regimens have been intensified or simplified by the addition or substitution of re-purposed or novel drugs with the goal of shortening the duration of therapy [7, 9]. Increasing public investment in the area has fostered the formation of large clinical trial networks and public-private partnerships (TB Alliance, PanACEA, ACTG/CDC TB Trials Consortium, PAN-TB, UNITE4TB)[1, 21, 22, 25, 26]. Efforts have also been made to encourage the sharing of clinical trial data through platforms such as TB-PACTS and public data calls from the World Health Organisation (WHO) with the goal of conducting large-scale individual patient data meta-analyses [4]. While the WHO has promoted standardised definitions of treatment outcomes for use by National TB Programmes [20, 27, 28], those used in clinical trials have not yet been formally harmonised by investigators.

While phase III trials typically focus on microbiological outcomes, their interpretation is dependent on the clinical context (for example whether associated with clinical symptoms and signs of disease, whether occurring on or after treatment, single versus repeated samples) and how they are incorporated into composite outcomes representing other “unfavourable” intercurrent events such as death, treatment discontinuation or modification, and loss to follow-up. There are current efforts to identify markers that predict efficacy (surrogate endpoints), although no marker has yet been identified, and a combination of outcomes reflecting bacterial and host factors is likely to be of value [19]. Empirical studies have identified substantial variability in the selection and reporting of III outcomes by clinical trialists ([2]), and the protocols of recent trials [12, 13].

Lack of consensus and harmonisation in defining the outcomes for clinical studies is problematic in synthesising evidence in traditional systematic reviews and individual patient data meta-analyses, which are the foundation of both policy and guideline development [19]. Furthermore, there is limited information on how they are valued by patients [14]. There is a need for future TB trials to produce high-quality evidence that supports policy decisions with optimal selection of endpoints and markers of treatment outcomes to provide meaningful results to clinicians, regulators, and guideline developers, who often must make recommendations based on low-certainty evidence [19].

An agreed standardised set of outcomes to measure in every phase III study of TB treatments would overcome these issues. A set of minimum outcomes that should be measured is known as a “Core Outcome Set”. While outcomes will not be restricted to those in the COS, it will be expected that data on core outcomes are collected and reported to enable effective evidence synthesis [6].

### Objectives

1. To compile a comprehensive list of candidate clinical, microbiological, radiological, and patient-reported outcomes based on systematic reviews of the published literature, an update of these systematic reviews, a review of active trials and protocols identified from clinicaltrials.gov, and from a qualitative survey of people being treated for pulmonary TB.
2. To achieve consensus on a COS for reporting outcomes of phase III clinical trials of treatment of pulmonary TB using remote surveys and consensus meetings.
3. To disseminate and promote the implementation of the phase III COS among key stakeholders.

### Scope

#### Condition

Pulmonary tuberculosis is confirmed by mycobacterial culture or the nucleic acid amplification test (NAAT), irrespective of the isolate’s susceptibility.

In most historical trials of TB treatment, confirmation of the target condition rested on positive identification of *M. tuberculosis* by culture methods. NAAT tests are now accepted as confirmation of pulmonary disease even if baseline cultures are subsequently negative [27, 28]. Patients with positive NAAT are at risk of poor longer-term outcomes based on culture-based assessments, even though NAATs are not recognised as a reliable means of monitoring outcome. Susceptibility of the infecting isolate, though it may determine the composition and duration of the regimen with which a person with TB is treated, does not in itself influence the methods by which efficacy and safety are assessed, so trials of people with drug-susceptible and drug-resistant disease will be included. Extra-pulmonary TB, including severe forms such as TB meningitis or pericardial TB, will be considered out of scope, as the clinical presentation differs substantially from pulmonary TB, and relevant trials typically focus on disease-specific outcomes rather than the pulmonary TB microbiological and clinical targeted in this COS. Similarly, latent TB infection will also be excluded from scope.

#### Population

Adults (≥16 years) receiving TB treatment.

The diagnosis of paediatric TB is typically not based on microbiological confirmation but on one of several scoring systems based on risk and clinical features. Case misclassification is an issue affecting clinical trials [9, 10], and hence, efficacy trials in TB are rarely conducted in children, though older children with pulmonary disease that is smear, culture, or NAAT positive have been included in some more recent trials.

#### Interventions

Combination regimens of antimicrobial agents, irrespective of the susceptibility of the isolate.

A COS developed for combination antibiotic regimens may also be applicable to host-directed therapies and therapeutic vaccination, but these interventions may require supplementary secondary or different primary outcomes pertinent to their therapeutic goals, for example measures of lung damage or prevention of re-infection. For this reason, they have not been included in the initial survey for the COS.

#### Setting

Phase III trials of TB treatment, whether efficacy or effectiveness trials and irrespective of susceptibility of the isolate or geographic setting.

Design innovations in TB trials in recent years mean that phase III endpoints are increasingly collected in a phase II context [23], while some traditional phase II endpoints, such as sputum culture conversion, are also readily measured during phase III trials, providing important information about the performance of these measures in predicting longer-term outcomes. Hence, consensus on outcomes in both contexts of use would be valuable.

## Methods

The study has been prospectively registered with the COMET database of core outcome sets (study 2195).

This protocol was developed according to the Core Outcome Set-STAndards for Development (COS-STAD) recommendations [16] and using the checklist outlined in the Core Outcome Set-STAndardised Protocol Items (COS-STAP) Statement [17].

### Study oversight and public involvement

A Study Steering Committee (SSC) will provide study oversight and advice. SSC members will be broadly representative of key stakeholder groups, including people with lived experience of TB, physicians, nurses, clinical trialists, statisticians, and policymakers (consumers of research outputs), taking into account internal support already available within the UNITE4TB consortium and inclusion of critical external partners. This mix of perspectives and skills is necessary to ensure that oversight and framing of COS processes and tools are broadly representative of the target stakeholder groups.

Members of the Study Steering Committee will be identified from existing networks. We aim to include members from a range of geographical locations who have a range of experiences linked to the stakeholder groups already described (Appendix 1).

The study’s day-to-day management will be the responsibility of a Study Management Group (GD, LB, RT, NH, PRW), which will meet regularly between formal SSC meetings to organise and administer the project.

### Identification of outcomes

The outcomes list used in the online consensus process will be generated through a review of outcomes used in clinical trials and those reported by patients.

Bonnett et al*.* previously published a systematic review of phase III pulmonary TB clinical trial outcomes [2]. We will update this review with added search terms for multi-drug-resistant TB and new TB treatments that have been through phase III trial. We will make sure the relevant regulatory documents are included in the review, including WHO policy documents. We note a distinction between the outcomes in clinical trials and programmatic outcomes and will include those relevant to trial outcomes only.

We will also review outcomes reported in protocols identified by Hills et al. [12, 13] and supplement this with a review of active trials identified from the clinicaltrials.gov registry. Outcomes will be extracted verbatim from the methods and results sections of eligible studies. We will also extract information on the method and timing of outcome assessment. The taxonomy of Dodd et al. will be applied to facilitate the grouping and review of outcomes [8]; verbatim outcomes will be reviewed and grouped into generic outcomes where the outcome is the same but worded differently.

Where composite outcomes are reported, we will record the disaggregated individual outcomes of which they are comprised. If a patient-reported outcome measure (PROM) is used, we will report all domains measured by that PROM.

Outcomes will also be annotated with the study phase in which they were measured, i.e. phase III (as stated by the authors), and whether the study is in DS—or DR-TB.

### Outcomes important to patients

Outcomes potentially important to patients relevant to phase III trials will be identified from three sources:

1. Domains measured in patient-reported outcome measures that have been used in the trial protocols [12, 13] and trial registry entries
2. Domains measured in quality-of-life measures identified from a systematic review by Khan et al. [15]
3. Outcomes identified from papers included in a qualitative synthesis of outcomes [14]

Outcomes will be extracted from direct quotes or summaries that relate to how a patient felt or functions or any experiences related to the treatment received for TB. The outcomes will then be applied to the COMET taxonomy of outcomes (Dodd 2016).

### The long list of outcomes

The outcomes identified from all sources will be combined to create a single long list of all possible outcomes for the core outcome set. Verbatim outcomes will be grouped into generic outcome names. This list of outcomes will be reviewed by the SSC, who will confirm that outcomes have been grouped appropriately, comment on the plain language descriptions, and add any outcomes they consider to be missing from the list**.** Should a large number of outcomes be identified, the source of the outcome, i.e. clinical trial or patient reported, and the number of studies reporting that outcome will be recorded. This information will be used in discussion with the SSC to agree on a manageable list of outcomes for the Delphi survey. Delphi participants will have the opportunity to add outcomes that they think are missing from the list. Any outcomes not taken forward to the Delphi survey will be recorded as outcomes.

The list of outcome measures will be defined in plain language. Initially, outcomes will be grouped into the domains of the COMET taxonomy described by Dodd et al., but these will be discussed with the SSC to determine suitability for use in the online Delphi survey.

### Consensus process

Consensus will be sought on which outcomes are critically important to measure in future trials (*what* outcomes) via a global Delphi survey. Researchers and healthcare workers will be asked:

“*When treating patients with pulmonary tuberculosis, what outcomes do you consider to be the most important to assess the effect of treatment?*”

People diagnosed with TB will be asked:

“*What outcomes are most important in helping you decide if your treatment is working for you? Important outcomes can also be ones that tell you your treatment is not working.*”

People in the TB civil society will be asked:

“*What outcomes are most important, within the community, when deciding if someone’s treatment is working for them or not?*”

### Stakeholder groups, recruitment, and eligibility

The diverse pool of stakeholders critical to the representativeness and dissemination of the COS will be invited through existing networks such as the UNITE4TB consortium and the SSC. People with lived experience of TB will be recruited via these networks and via community gatekeepers. Participants will be eligible to take part if they are over 18 years of age, provide informed consent, and are in one of the following stakeholder groups:

1. Researchers (including trialists and statisticians) working in either academia or industry.
2. Health professionals, including clinicians, laboratory clinicians, specialist nurses, community health workers, and allied medical professionals.
3. Anyone who has been diagnosed with TB and carers of people who have been diagnosed.
4. Working in the TB community/civil society locally, regionally, nationally, or internationally. Working with people at risk of TB, TB survivors, TB advocates, local community groups, families affected by TB, Community Action Boards (CABs), working with key and vulnerable populations, and working in human rights.

Regulators and policymakers will not participate in the consensus process as a stakeholder group. However, their engagement is important in promoting the endorsement and uptake of the COS, and representatives from regulatory bodies such as the US Food and Drug Administration (FDA) and European Medicines Agency (EMA) will be invited to comment on the COS and participate in its subsequent implementation. The Study Management Group will engage with these groups separately to discuss the study, including the opportunity to suggest any important policy outcomes that may be missing from the list.

Upon registration to take part in the Delphi survey, participants will be asked to provide details relating to their role and stakeholder group. The stakeholder eligibility criteria are detailed in Appendix 2.

The consensus process will be managed online using Delphi Manager software (COMET Initiative UK) [5]. All participants will be asked to register to take part and to provide details of their country of residence, gender, age, and stakeholder group. People with lived experience of TB will be asked how long ago they concluded their treatment or if it is ongoing. Healthcare professionals and researchers will be asked for details on their role and years of experience in caring for people with TB. The TB community members will be asked about what kind of community organisation they belong to and whether they work at an international, national, or local level. Information about what taking part involves will be provided prior to registration, and consent will be sought online, with participants confirming that they have read and understood the information and consent to take part. All materials will be restricted to the English language; any written materials will be presented to the patient-public representatives in the SSC to ensure they are in plain English and understandable to a diverse group of English-speaking stakeholders.

#### Round 1

In the first round of the Delphi survey, participants will be presented with a list of outcomes, with a plain language summary of each. The SSC will contribute to the development of the plain language descriptions. Participants will be asked to rate each outcome in the list using the Grading of Recommendations, Assessment, Development and Evaluations (GRADE) nine-point Likert scale [11] with ratings of 1–3 denoting outcomes not that important, 4–6 outcomes that are important but not critical to measure in every trial, and 7–9 outcomes that are critically important to measure in every trial. There will also be an option of “unable to rate” and to provide free-text feedback on individual outcomes.

Outcomes will be presented grouped by domain and the domain order randomised to help avoid presentation bias.

After rating all outcomes, participants will have the option to add any additional outcomes they think are critically important but missing from the list. Free-text responses will be reviewed and discussed by the SSC. Any new outcomes identified will be added to the list in round 2.

#### Round 2

Participants who have rated 50% or more of the outcomes in round 1 will be invited to take part in round 2.

All round 1 outcomes will be taken forward to round 2, together with any new outcomes identified. A summary of the results from round 1 will be presented graphically for each outcome, together with an individual’s own rating. Participants will be asked to consider this information before rating the outcome again.

At the end of round 2, the number of participants in each stakeholder group rating each value 1–9, for each outcome, will be summarised, and the definition of consensus will be applied (Table 1).

**Table 1:** Consensus definitions

Consensus classification	Description	Definition
Consensus in	Consensus that outcome should be included in the core outcome set	80% or more participants in each stakeholder group scoring as 7 to 9 and <10% participants scoring as 1 to 3
Consensus out	Consensus that outcome should not be included in the core outcomes set	50% or less participants in each stakeholder group scoring as 7 to 9
No consensus	Uncertainty about importance of outcome	Anything else

#### Consensus meeting

**Fig. 1:**
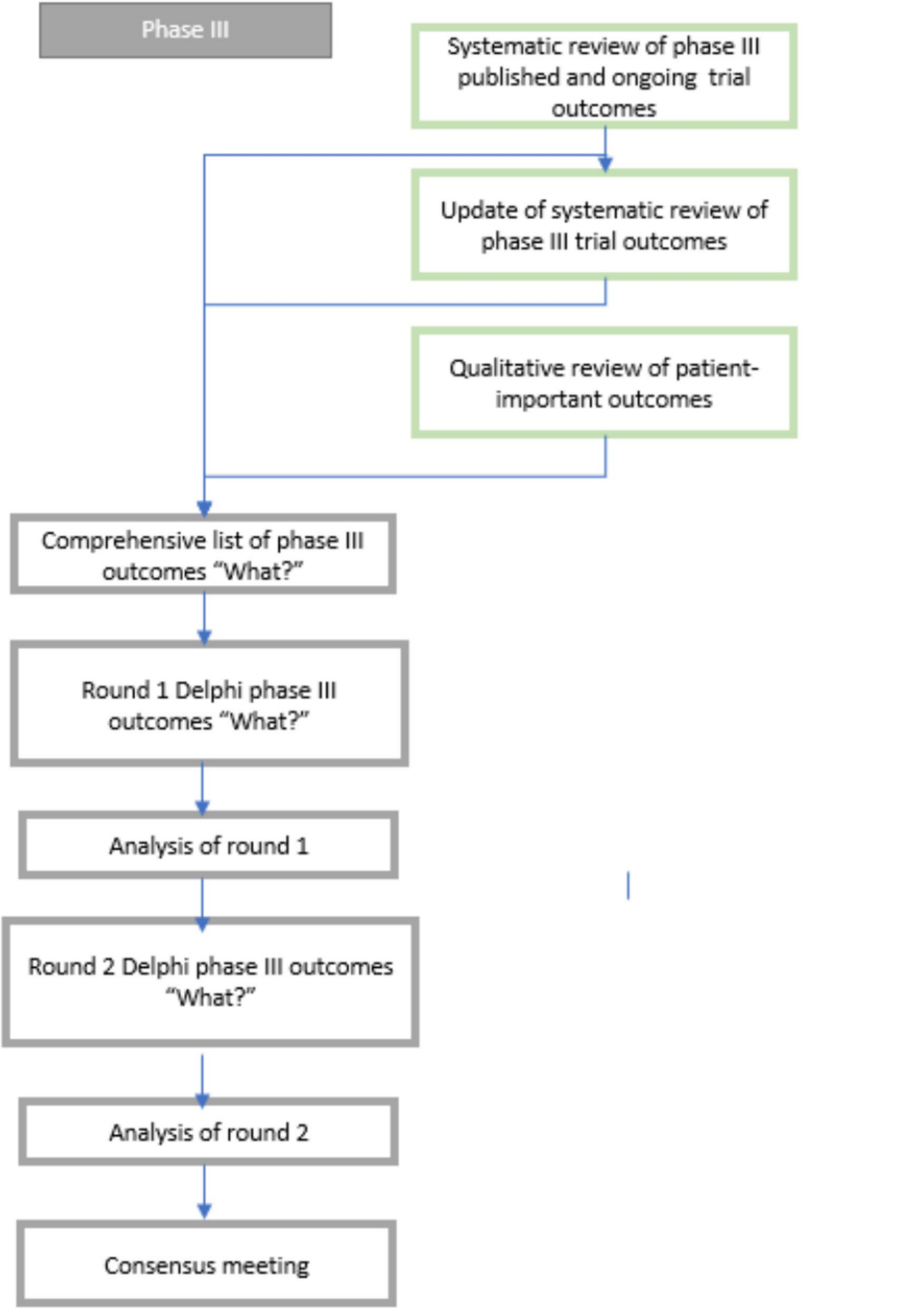
Flow diagram of data-gathering and consensus process

We will hold an online consensus meeting to discuss the results of the Delphi survey and agree on a core outcome set. Participants who took part in both rounds of the Delphi survey will be invited to express their interest in attending the meeting. The SSC will review the expressions of interest, and invitations will be issued to ensure suitable representation of all stakeholder groups at the consensus meeting (Fig. 1).

### Consensus definition

We will apply the definition of consensus (Table 1) to the results of the Delphi survey. There is no sample size calculation, but we will aim for a minimum of 10 participants per stakeholder group.

Outcomes that meet the definition of “consensus in” after round 2 of the Delphi will be included in the COS; outcomes that meet the definition of “consensus out” will be excluded. Outcomes with no consensus will be prioritised for discussion in the online consensus meeting. The consensus meeting will involve discussion of the outcomes with no consensus, followed by anonymous rating using the 1–9 rating scale. The consensus definitions in Table 1 will be applied. Where there is no consensus, the outcome will not be included in the COS. A consensus report will be written that describes the discussion and results of rating for each outcome discussed at the consensus meeting.

### Missing data

We will record the proportions of complete responders, partial responders, and non-responders at each stage of the Delphi process. We will encourage participants who only partially complete round 1 to complete the survey in full, and those who complete 50% or more of the survey will be invited to round 2. Bias from the loss of participants due to partial or non-response between rounds will be assessed by determining if there is any difference in median round 1 scores between those who have completed both rounds and those who only completed round 1.

## Ethics and dissemination

### Ethics approval/informed consent

Ethical approval has been received from the Central Research Ethics Committee of the University of Liverpool (REC reference 12254). Written study information will be provided to participants, making clear that their participation is voluntary, and informed consent will be sought online using Delphi Manager prior to participants accessing the outcomes list. Participants who have completed both rounds of the Delphi survey and expressed an interest in attending the consensus meeting will be provided with meeting information, and those who join the meeting will be considered to have provided implied consent by attending. The outcomes list will be reviewed for items that may potentially cause participant distress, particularly to people with lived experience of TB. If this does occur, participants will be advised to contact their current TB team or their general practitioner, as well as appropriate TB support groups.

### Dissemination and application

The study has been registered with the COMET Initiative database (Study ref. 2195). We plan to disseminate the findings of the COS process to the major stakeholders identified above and to the participants in the surveys and meetings. The results will be published in an open-access peer-reviewed journal, with links to the location of the available anonymised data set. The COS will be presented in relevant research forums. A plain language summary of the work will also be shared.

The COS will form part of a package also comprising a linked estimands framework and updated data standard specification which is intended to be applicable to all trials of TB treatment within and outside of UNITE4TB’s activities. These outputs will be promoted for adoption by drug developers, clinical trial networks, and guideline groups such as WHO.

Application of the COS and limitations will be included in the manuscript detailing the development of the COS. This will allow full consideration of limitations based on actual versus planned activities.

## Administrative information

### Funders

This work is funded by the UNITE4TB consortium, a public-private partnership funded by the Antimicrobial Resistance Accelerator of the European Union Innovative Medicines Initiative. Relevant disclosures and disclaimers will be monitored by the SSC and attached to relevant publications and presentations.

### Trial status

Recruiting (27/11/23–30/04/2025).

## Data Availability

The anonymised dataset will be available upon request from the corresponding author.

## Appendix 1

**Table 2:** Study steering committee

Name	Affiliation	Country
Patrick Phillips	Associate Professor, Statistician, Center for Tuberculosis, University of California San Francisco	USA
Hanif Esmail	Clinical Associate Professor, University College London, Medical Research Council Clinical Trials Unit	UK
Daniela Cirillo	Clinical Microbiologist, Head of the Emerging Bacterial Pathogen Research Unit, Deputy Director, Division of Immunology, Transplant and Infectious Diseases, IRCCS San Raffaele Scientific Institute	Italy
Susan Dorman	Professor, Infectious Disease Specialist and TB researcher, Medical University of South Carolina	USA
Christian LeInhardt	Infectious Disease Specialist and Clinical Epidemiologist, Emeritus Research Director French Institute for Research and Sustainable Development (IRD)	France
Jonathan Stillo	Associate Professor, Medical Anthropologist, Wayne State University	USA
Oxana Rucsineanu	Executive Director, Moldova National Association of Tuberculosis Patients	Moldova
Blessina Kumar	Co-Founder and CEO Global Coalition of TB Activists	India
Carrie Tudor	Deputy Director, SMART4TB John Hopkins School of Medicine, Research Associate, John Hopkins School of Nursing	South Africa
Katie Rolfe	Statistics Director, GSK Global Health Group	UK
Jeremiah Chakaya	Professor of Global Respiratory Health, Liverpool School of Tropical Medicine, Technical Director, Respiratory Society of Kenya	UK, Kenya

## Appendix 2

### Stakeholder eligibility criteria and recruitment

#### Researchers (including trialists and statisticians)

Researchers in the field of TB treatment, including clinical trialists and statisticians. Recruitment via networks of the SSC including the core UNITE4TB team and other relevant UNITE4TB WPs. Other key partners will include:

- International Union Against Tuberculosis and Lung Disease
- CDC TB Trials Consortium [3]
- PAN-TB Consortium [22]
- PANACEA consortium [21]
- European Lung Foundation
- American Thoracic Society
- ACTG [1]

#### Health professionals

TB physicians and nurses with experience caring for people with TB and managing their treatment will be recruited via the SSC’s networks, including the core UNITE4TB team and other relevant UNITE4TB WPs.

#### People with lived experience of TB

People 18 years of age or older, currently on treatment or previously treated for clinically or microbiologically confirmed drug-susceptible or drug-resistant TB. The project team will be aware of whether the participant’s treatment has been concluded, but this does not preclude the participant’s ability to participate. Recruitment will be via networks of the UNITE4TB Community Advisory Board and other patient advocacy organisations. Efforts will be made to ensure stakeholders from underrepresented groups, such as women of reproductive age, are present.

#### Tuberculosis civil society/community members

In response to feedback from the initial round of recruitment, those not directly affected by TB but are part of the TB civil society/community will be included as a distinct group. They will be asked:

- Are you involved on the tuberculosis community locally, regionally, nationally, or internationally? (choose one)
  i. I am involved in my local area.
  ii. I am involved in my region.
  iii. I am involved at the country level.
  iv. I am involved at the international level.
- What kind of community or civil society group are you involved with? (click all that apply)
  o People at risk of tuberculosis
  o People who are receiving TB treatment
  o People who have survived tuberculosis
  o Tuberculosis advocates
  o Local community group
  o Families of people affected by tuberculosis
  o Community Advisor Board
  o Vulnerable and key populations
  o Human rights organisation
  o Other (free text)

## References

[1] ACTG. https://www.cdc.gov/tb/research/tbtc.html. Accessed 20th May 2024.

[2] Bonnett LJ, Ken-Dror G, Davies GR. Quality of reporting of outcomes in phase III studies of pulmonary tuberculosis: a systematic review. Trials. 2018;19:134. 10.1186/s13063-018-2522-x. 29467027 10.1186/s13063-018-2522-x PMC5822642

[3] CDC TB Trials Consortium. https://www.cdc.gov/tb/research/tbtc.html. Accessed 20th May 2024.

[4] Collaborative Group for the Meta-Analysis of Individual Patient Data in MDR-TB treatment–2017. Treatment correlates of successful outcomes in pulmonary multidrug-resistant tuberculosis: an individual patient data meta-analysis. Lancet. 2018;392(10150):821–834. 10.1016/S0140-6736(18)31644-1 PMC6463280 30215381

[5] COMET initiative UK, Delphi manager. https://www.comet-initiative.org/delphimanager/index.html. Accessed 20 May 2024.

[6] COMET initiative UK, overview. https://ctrc.liv.ac.uk/InDevelopment/Websites/COMET/about/overview. Accessed 20 May 2024.

[7] Conradie F, Diacon AH, Ngubane N, Howell P, Everitt D, Crook AM, et al. Treatment of highly drug-resistant pulmonary tuberculosis. N Engl J Med. 2020;382:893–902. 32130813 10.1056/NEJMoa1901814 PMC6955640

[8] Dodd S, Clarke M, Becker L, Mavergames C, Fish R, Williamson PR. A taxonomy has been developed for outcomes in medical research to help improve knowledge discovery. J Clin Epidemiol. 2018;96:84–92. 29288712 10.1016/j.jclinepi.2017.12.020 PMC5854263

[9] Dorman SE, Nahid P, Kurbatova EV, Phillips PPJ, Bryant K, Dooley KE, et al. Four-month rifapentine regimens with or without moxifloxacin for tuberculosis. N Engl J Med. 2021;384:1705–18. 33951360 10.1056/NEJMoa2033400 PMC8282329

[10] Guglielmetti L, Khan U, Velásquez GE, Gouillou M, Abubakirov A, Baudin E, Berikova E, Berry C, Bonnet M, Cellamare M, Chavan V, Cox V, Dakenova Z, de Jong BC, Ferlazzo G, Karabayev A, Kirakosyan O, Kiria N, Kunda M, Lachenal N, Lecca L, McIlleron H, Motta I, Toscano SM, Mushtaque H, Nahid P, Oyewusi L, Panda S, Patil S, Phillips PPJ, Ruiz J, Salahuddin N, Garavito ES, Seung KJ, Ticona E, Trippa L, Vasquez DEV, Wasserman S, Rich ML, Varaine F, Mitnick CD; endTB Clinical Trial Team. Oral regimens for rifampin-resistant, fluoroquinolone-susceptible tuberculosis. N Engl J Med. 2025;392(5):468–482. 10.1056/NEJMoa2400327 PMC7617355 39879593

[11] Guyatt GH, Oxman AD, Kunz R, et al. Grade guidelines: 2. Framing the question and deciding on important outcomes. J Clin Epidemiol. 2011;64(4):395–400. 21194891 10.1016/j.jclinepi.2010.09.012

[12] Hills NK, Lyimo J, Nahid P, Savic RM, Lienhardt C, Phillips PPJ. A systematic review of endpoint definitions in late phase pulmonary tuberculosis therapeutic trials. Trials. 2021;22:515. 10.1186/s13063-021-05388-1. 34344435 10.1186/s13063-021-05388-1 PMC8329622

[13] Hills NK, Phillips P, Weir I. The specification of estimands in pulmonary TB treatment clinical trials: a proposal for improving standardization of endpoints and handling of intercurrent events utilizing the ICH E9 (R1) Addendum Framework 2021 (unpublished).

[14] Hoppe LE. Developing core outcome sets for clinical research and guideline development – qualitative systematic reviews to increase the volume, depth and diversity of patient perspectives included. Tuberculosis: a case study (MPhil Thesis). 2016. Available at: https://www.research.manchester.ac.uk/portal/files/54586722/FULL_TEXT.PDF.

[15] Khan S, Tangiisuran B, Imtiaz A, Zainal H. Health status and quality of life in tuberculosis: systematic review of study design, instruments, measuring properties and outcomes. Health Serv J. 2017;11:484–94.

[16] Kirkham JJ, Davis K, Altman DG, Blazeby JM, Clarke M, Tunis S, et al. Core outcome set-STAndards for development: the COS-STAD recommendations. PLoS Med. 2017;14:e1002447. 29145404 10.1371/journal.pmed.1002447 PMC5689835

[17] Kirkham JJ, Gorst S, Altman DG, Blazeby JM, Clarke M, Tunis S, Williamson PR; COS-STAP Group. Core outcome Set-STAndardised protocol items: the COS-STAP statement. Trials. 2019; 20:116. 10.1186/s13063-019-3230-x PMC6371434 30744706

[18] Knight GM, McQuaid CF, Dodd PJ, Houben RMGJ. Global burden of latent multidrug-resistant tuberculosis: trends and estimates based on mathematical modelling. Lancet Infect Dis. 2019;19:903–12. 31281059 10.1016/S1473-3099(19)30307-X PMC6656782

[19] Lienhardt C, Vernon A, Cavaleri M, Nabiar S, Nahid P. Development of new TB regimens: harmonizing trial design, product registration requirements, and public health guidance. PLoS Med. 2019;16(9):e1002915. 31490921 10.1371/journal.pmed.1002915 PMC6730844

[20] Linh NN, Viney K, Gegia M, Falzon D, Glaziou P, Floyd K, et al. World Health Organization treatment outcome definitions for tuberculosis: 2021 update. Eur Respir J. 2021;58:2100804. 34413124 10.1183/13993003.00804-2021

[21] PanACEA. panacea-tb.net. Accessed 20th May 2024.

[22] PAN-TB. https://www.pan-tb.org/. Accessed 20th May 2024.

[23] Phillips PP, Dooley KE, Gillespie SH, Heinrich N, Stout JE, Nahid P, et al. A new trial design to accelerate tuberculosis drug development: the phase IIC selection trial with extended post-treatment follow-up (STEP). BMC Med. 2016;14:51. 10.1186/s12916-016-0597-3. 27004726 10.1186/s12916-016-0597-3 PMC4804526

[24] Review on Antimicrobial Resistance. Tackling drug-resistant infections globally: final report and recommendations. 2016. https://amr-review.org/. Accessed 20^th^ May 2024.

[25] TB Alliance. https://www.tballiance.org/. Accessed 20th May 2024.

[26] UNITE4TB. https://www.unite4tb.org/. Accessed 20th May 2024.

[27] WHO. Global tuberculosis report 2024 global. Geneva: World Health Organization; 2024. Licence: CC BY-NC-SA 3.0 IGO. https://iris.who.int/bitstream/handle/10665/379339/9789240101531-eng.pdf?sequence=1. Accessed July 2025.

[28] WHO. WHO consolidated guidelines on tuberculosis. Module 3: diagnosis – rapid diagnostics for tuberculosis detection, third edition. Geneva: World Health Organization; 2024. Licence: CC BY-NC-SA 3.0 IGO. https://iris.who.int/bitstream/handle/10665/376221/9789240089488-eng.pdf?sequence=1. Accessed 08th July 2024.

[29] WHO. WHO consolidated guidelines on tuberculosis. Module 4: treatment and care. Geneva: World Health Organization; 2025. Licence: CC BY-NC-SA 3.0 IGO. https://iris.who.int/bitstream/handle/10665/380799/9789240107243-eng.pdf?sequence=1 Accessed July 2025. 40163610

